# Myasthenia Gravis With Thymoma, Manifesting as AChR-Ab-Positive, Distinct Bulbar Palsy Accompanied by Dysgeusia: A Case Series and Review of Literature

**DOI:** 10.3389/fneur.2018.00214

**Published:** 2018-04-04

**Authors:** Kai Zhu, Jiaxin Chen, Jingjing Li, Haiyan Wang, Xin Huang, Yan Li, Huiyu Feng

**Affiliations:** ^1^Department of Neurology, The First Affiliated Hospital of Sun Yat-sen University, Sun Yat-sen University, Guangzhou, China; ^2^Guangdong Provincial Key Laboratory for Diagnosis and Treatment of Major Neurological Disease, Guangzhou, China; ^3^Department of Neurosurgical Intensive Care Unit, The First Affiliated Hospital of Sun Yat-sen University, Sun Yat-sen University, Guangzhou, China

**Keywords:** myasthenia gravis, dysgeusia, antibody positive, thymoma, bulbar palsy

## Abstract

In this review, we summarized three cases of myasthenia gravis (MG) with taste disorder and describe their clinical features in detail. Three MG patients presented with significant bulbar palsy symptoms, high AChR-Ab titers, and negative MuSK-Ab, were diagnosed with thymoma. Furthermore, we observed that dysgeusia could manifest earlier than the occurrence of typical MG symptoms, even predict a MG relapse or a myasthenic crisis in the course of MG. We believe that dysgeusia is a non-motor symptom of MG, which especially exists in MG patients with thymoma and serious bulbar palsy. Therefore, being alert to this symptom may facilitate the early diagnosis of MG and judge the progress of the disease.

## Introduction and Background

Myasthenia gravis (MG) is an AChR-Ab-mediated autoimmune disease affecting the neuromuscular joints. Typical clinical manifestations include fluctuating weakness of extraocular muscles or skeletal muscles. The symptoms are usually aggravated by activity and relieved by rest (1). The muscles involved include extraocular muscles, pharyngeal muscles, laryngeal muscles, muscles of the extremities, and respiratory muscles. According to literature, non-motor symptoms occur in about 25% of MG cases, including alopecia areata, pure red cell aplasia, and dysgeusia (2). According to current literature, MG with taste disorder primarily manifests as a decrease or loss of sweet taste (3–6) and is associated with abnormal T-cell immune tolerance induced by the thymoma (2). In our study, taste was tested by the taste strips method. Of the 412 MG patients who were followed-up, three were associated with dysgeusia (incidence rate: 0.7%). The clinical data of the three cases are summarized below with a review of the relevant literature.

## Case Presentation

### Patient 1

A 55-year-old man was diagnosed with MG in April 2007 owing to repeated episodes of blepharoptosis with diplopia. Results of the neostigmine test were positive. The patient also tested positive for single-fiber electromyography (SFEMG) and repetitive nerve stimulation (RNS). AChR-Ab was 45.92 nmol/L and negative for the presence of MuSK-Ab.

The patient had a long medical history and presented with complicated conditions. Around October 2006, 6 months before the confirmed diagnosis of MG, the patient experienced dysgeusia, with the manifestations of loss of taste (failure to recognize all kinds of tastes such as sweet, sour, bitter, and salty). His olfaction was normal, and he visited the Otolaryngology and Stomatology Departments of a local hospital, where no significant abnormality was detected. Further detailed examinations such as cranial magnetic resonance carried out at the Neurology Department also did not show any obvious abnormality. Only the results of the taste strips method indicated that he suffered complete ageusia, with the most noticeable loss in taste of sweetness at the apex of the tongue (7). No confirmed diagnosis was made. At that time, the patient had no history of diabetes and hypertension, but he had a smoking history of over 10 years, at the rate of 35 pack years. With stable taste disorder symptoms, the patient did not show any obvious exacerbation or remission. However, after 6 months of dysgeusia, the patient experienced ptosis, with mild symptoms in the morning that became severe by evening, and which was significantly aggravated after fatigue. The patient again visited the Neurology Department owing to the obvious symptoms of MG. After further relevant examinations, a confirmed diagnosis of MG was made in April 2007. Following this, the patient started to receive cholinesterase inhibitor (pyridostigmine bromide) and immunosuppressor (prednisone) therapy. Thereafter, the symptoms of blepharoptosis improved, but no obvious improvement or fluctuation in clinical symptoms was observed with respect to the dysgeusia. In August 2007, the patient underwent a thymic computed tomography, during which concomitant thymoma was detected. In October of the same year, the patient underwent thymectomy. The procedure was successful, and postoperative thymus pathology indicated thymoma type B1, Masaoka stage I. One week after the operation, his dysgeusia spontaneously resolved. He could differentiate savory, sweet, sour, bitter, and salty tastes. A follow-up test of AChR-Ab titer was 21.30 nmol/L, which was remarkably lower than the previous value. Even after persistent postoperative treatment with cholinesterase inhibitor, hormones, and immunosuppressants, his muscle weakness fluctuated and was aggravated following a common cold infection. Following the surgery, the patient had five episodes of myasthenic crisis. Because of involving pharyngeal and laryngeal muscles, at his worst, the patient was on a nasogastric tube for over one and a half years. However, the dysgeusia never recurred after the operation, even 10 years later (as of August 2017).

### Patient 2

A 27-year-old woman was diagnosed with MG (MGFA Type IIb) in January 2017, owing to 2 months of ptosis accompanied by extremity fatigue and dysphagia. The patient started to have dysgeusia in December 2014, which mainly manifested as loss of sweet taste, most remarkable at the tip of the tongue. Other tastes such as sourness, bitterness, and saltiness were normal. Her sense of smell was normal. As it did not affect her daily life, it was not taken seriously, nor was it diagnosed or treated. The patient had no history of diabetes, hypertension, smoking, or drinking. It is worthwhile to note that she became pregnant in January 2016. During her pregnancy, she had regular prenatal examinations and, on October 11, 2016, successfully gave birth to a baby boy. Despite the uneventful pregnancy, the loss of sweet taste persisted. Around November 20, 2016, the patient developed left ptosis and diplopia. This symptom aggravated at night but was relieved in the morning and aggravated after activity but relieved after rest. Although she presented to the Ophthalmology Department, the diagnosis was not clear. About a month later, the symptoms progressed to bilateral ptosis, with the same characteristics as above. Examination in the outpatient service of the Neurology Department showed that the patient had a positive neostigmine test. RNS shows that right facial and bilateral accessory nerves had decreasing amplitudes under both low- (5 Hz) and high- (15 Hz) frequency stimulation (about 14 and 32%, respectively). AChR-Ab was 25.73 nmol/L and anti-MuSK antibody titer was negative. She was diagnosed with MG. Following treatment using pyridostigmine bromide 60 mg t.i.d., the patient’s ptosis and diplopia improved with no significant improvement in the loss of sweet taste. In December 2016, she experienced weakness of upper limbs after physical exertion. The symptoms were slightly relieved after increasing the pyridostigmine bromide dose to 90 mg t.i.d., but no obvious change was noted in the dysgeusia. On January 20, 2017, the patient developed slurred speech, unclear articulation, masticatory insufficiency, and dysphagia. Therefore, she could only consume semi-liquid diets such as porridge or rice paste. The loss of sweet taste was unchanged. The neurologist considered an exacerbation of her MG symptoms. Subsequently, the patient was given intravenous injections of high-dose immunoglobulin (gamma globulin 0.4g/kg per day) for three consecutive days and prednisone 2.5 mg q.d. for immunosuppression therapy. After 7 days, the dose of prednisone was increased to 5 mg and then to 10 mg after 3 weeks. The patient’s symptoms of ptosis, limb weakness, masticatory atonia, and dysphagia were significantly relieved. However, the loss of sweetness still remained unchanged. On April 18, 2017, she underwent a thymic CT examination, which revealed a nodular soft tissue shadow anterior to the main pulmonary artery of the anterior mediastinum. Consequently, on May 4, 2017, the patient underwent extended thymectomy under general anesthesia. The procedure was uneventful, and postoperative pathology indicated thymoma type B2, Masaoka Stage 1. After the operation, the patient’s ptosis, limb weakness, chewing, and swallowing symptoms were significantly relieved. However, she still experienced diplopia, and the loss of sweetness remained unchanged. Prednisone administration was continued and azathioprine was additionally administered. Approximately 2 months after the operation (in August 2017), gustometry was performed on the patient using the taste strips method (7). The results remained unchanged from pre- to postoperative stage.

### Patient 3

A 50-year-old man experienced left ptosis with diplopia in late January 2015. Subsequently, he experienced dysphagia and coughing while drinking water. Hence, he visited the Neurology Department for diagnosis and treatment. The patient had a positive neostigmine test, in addition to a positive RNS and SFEMG. The AChR-Ab titer was 17.40 nmol/L, and anti-MuSK antibody titer was negative. Therefore, he was diagnosed with MG. The symptoms improved after the administration of pyridostigmine bromide and prednisone, but fluctuation still occurred occasionally. At this time, he had no symptoms of dysgeusia. Magnetic resonance imaging performed in April 2015 revealed thymoma. In August 2015, the patient underwent thymectomy, and the postoperative pathology confirmed the thymoma to be of B1 and B2 mixed type, Masaoka Stage 1. A month later, the patient’s myasthenia symptoms (dysphagia and coughing while drinking) disappeared completely, but the left ptosis and diplopia still remained. Nine months after the operation in May 2016, in addition to the improvement in pharyngeal and laryngeal muscles, the patient’s diplopia disappeared, with occasional left ptosis. However, his reexamination showed that the AChR-Ab titer was 29.41 nmol/L, showing a significant increase as compared with pre-operative values. In June 2017 (14 months after the operation), the patient began to have dysgeusia, with the specific manifestation of loss of sweet taste at the apex of the tongue. No significant abnormality was detected in either olfaction or other tastes. One month later, he experienced an aggravation in myasthenia symptoms, with the manifestation of coughing while drinking, dysphagia, limb weakness, and respiratory difficulties. The patient was clinically diagnosed with impending myasthenic crisis. He was then given intravenous injections of high-dose immunoglobulin. Through standardized treatment, the patient’s symptoms improved evidently. In the meantime, dysgeusia was completely relieved. The gustometry performed on the patient using the taste strips method showed no abnormality.

## Discussion

Dysgeusia, specifically the decrease or loss of sweet taste, in patients with MG is still only poorly understood. It is hypothesized that the antibody generated by autoimmunity blocks the cholinergic receptors of the central nervous system. A number of studies have also confirmed this hypothesis by detecting the presence of acetylcholinergic receptors in the gustatory bud cells (8). Moreover, an animal-model experiment has shown that acetylcholine mediates the conduction of information between gustatory bud recipient cells and increases the activities of gustatory afferent neurofibers (9). Another hypothesis states that patients with MG generate a certain autoantibody that can act on the α-gustducin positive receptor, thereby interfering with the taste signal conduction pathway and further leading to ageusia (3). A multi-centric study from Japan has found that 4.3% of MG patients suffer from symptoms of dysgeusia. It is noteworthy that these patients also suffer from thymoma. The dominant symptom is loss in sweetness taste; in some cases, the symptoms of taste disorder are associated with the disease severity as well as the AChR-Ab titer; however, dysgeusia improved after immunotherapy or relapsed with the recurrence of thymoma (5). MG treatment drugs can often improve neurotransmission, and the use of hormones can improve the chemoreception functions of olfaction and gestation (4).

The three MG patients associated with dysgeusia in our study had similar characteristics to those reported in previous studies (Table 1): generalized MG, AChR-Ab-positive MG, and MG with thymoma. Patient 1 in our study had an AChR-Ab antibody titer of 45.92 nmol/L during the early onset stage. Following thymectomy, the antibody titer decreased to 21.30 nmol/L after the gustation recovered to normal. Patient 3 achieved complete remission from myasthenia symptoms for a certain period of time after the thymectomy. Subsequently, following symptom recurrence, a reevaluation of AChR-Ab revealed an increase in titer from 17.40 to 29.41 nmol/L. These suggest that MG with dysgeusia is related to the AChR-Ab titer. Of the three patients, patient 1 had the highest antibody titer, indicating the most severe gustatory impairment, with the manifestation of ageusia. This patient could not regain any of the gustatory senses. Patients 2 and 3 with a relatively low antibody titer also had relatively mild clinical symptoms, with the manifestation of solitary ageusia. Consistent with previous literature reports, loss in sweet taste was the major feature of MG with dysgeusia. Patient 3 immediately experienced a relapse of muscle weakness symptoms and progressed to impending myasthenic crisis after developing dysgeusia symptoms, and the serum antibody titer increased compared to earlier levels. The findings suggest that dysgeusia in these three patients was somewhat connected with MG. It can occur before the appearance of typical MG symptoms, also at the early stage of a myasthenic crisis in the course of MG. Therefore, we predict that dysgeusia in MG is associated with the changes in MG conditions and the severity may be related to the AChR-Ab titer. A possible exacerbation in the patient’s conditions should be considered if an MG patient has the chief complaint of apparent dysgeusia during clinical follow-up.

**Table 1:** Case reports of taste disorder associated with myasthenia gravis (MG).

Reference	Type	Country	Number of the taste disorder patients (gender)	Thymoma	Serum titers of AChR-Ab	Taste disorder			
						
						Preceding(−)/after(+) MG onset	Ageusia/hypogeusia/dysgeusia (sweet/salt/bitter/acid)	Response to MG treatment	Response to thymectomy (time after the operation)
Musha et al. (10)	Case reports	Japan	2 (1M, 1F)	+	+ (P1: 26.0 pmol/dL, P2: 16.2 pmol/dL)	P1: − (2 years)P2: − (5 years)	P1: hypogeusia (sweet and salt)P2: hypogeusia (sweet and salt)	P1: NDP2: recovery	

Sera et al. (11)	Case report	Japan	1 (F)	+	+ (118.0 nM, normal, <0.2)	At the same time	Hypogeusia (ND)	Relapse with MG crisis, relieved by plasmaphersis	Relapse with thymoma recumence

Kanzato et al. (12)	Case report	Japan	1 (M)	+	+ (100 nmol/L)	+ (ND)	Ageusia (ND)	Relapse with MG crisis	No changed

Takamori et al. (13)	Case report	Japan	1 (F)	+	+ (0.9 nmol/L normal, <0.6 nmol/L)	− (6 months)	Dysgeusia (sweet)	No changed	Improve (6 months)

Iseki et al. (6)	Case report	Japan	1 (M)	+	+ (75 nmol/L)	− (Several months)	Ageusia (sweet), hypogeusia (other taste modalities)	ND	Improve (3 days)

Nakazato et al. (14)	Case report	Japan	1 (M)	+	+ (43.0 nmol/L normal, <0.2 nmol/L)	− (8 years)	Ageusia (sweet)	ND	No changed

Kabasawa et al. (5)	Short communication	Japan	9 (2M, 7F)	+	ND	5 of 9: precedingOther: after		5 of 9: improveOther: ND	

*M, male; F, female; ND, no described*.

In all three cases, patients had obvious symptoms of bulbar palsy, and were negative for MuSK-Ab, which has not been mentioned in previous reports. Particularly in case 1, even with regular immunosuppressant drug therapy after thymoma removal, the patient still needed to rely on nasogastric tube feeding for as long as 1.5 years. We speculate that high titer of AChR-Ab leads to damage in lingual, pharyngeal, and laryngeal muscles (including AchR in taste buds); therefore, these patients show clinical symptoms similar to bulbar palsy in anti-MuSK-Ab positive MG. A thorough understanding of taste disorder in MG patients will help distinguish between different MG subtypes mediated by different antibodies.

Non-motor symptoms, unlike the symptoms of extraocular muscles, pharynx, and larynx muscles, rarely severely affect the daily life of an MG patient. However, dysgeusia also causes distress in a patient’s life. Dysgeusia often causes lower appetite and general unhappiness. Malnutrition caused by lower appetite is one of the chief reasons leading to poor quality of life. In addition, in MG patients, dysgeusia-induced emotional problems and unhappiness will increase mental burden affecting disease stabilization and control. Thus, attention to dysgeusia will help systemic diagnosis and treatment. In clinical practice, diagnosing dysgeusia in patients as early as possible will effectively avoid patients from ingesting harmful foods. For example, patients with sweet taste deficiency can be prevented from ingesting spoiled food or ingesting excess amounts of sugar that could lead to metabolic disorder resulting in central obesity due to fat redistribution. Sweet taste deficiency in patients likely causes excess sugar intake, combined with side effects from glucocorticoids, subsequently resulting in a series of health issues such as diabetes, hypertension, and coronary heart diseases, and affects the functions of multiple organs.

Unfortunately, dysgeusia is easily overlooked in the clinic. Medical staff seldom examine a patient’s gustatory senses during the usual physical examination. Chinese people commonly eat salty food and MG patients mostly present with the manifestation of the loss of sweet taste. Therefore, patients find it difficult to provide an accurate medical history of dysgeusia experienced in daily life, or they believe that dysgeusia does not compromise their quality of life and choose to ignore it, further delaying diagnosis and treatment. The multiple diseases concomitantly suffered by a patient are prone to be mistaken as the adverse reactions of other drugs or immunosuppressors and are wrongly interpreted. For example, azathioprine, methotrexate, and nifedipine can induce dysgeusia, the drug-induced gustatory function disorder (15).

## Concluding Remarks

As a non-motor symptom of MG, dysgeusia may assist us in learning the pathogenesis of MG with thymoma, and even guide MG diagnosis and judge its prognosis. The three cases of dysgeusia in this case report have common clinical characteristics: the obvious symptoms of bulbar palsy, high titer of AChR-Ab, negative MuSK-Ab, and thymoma. In addition, these three cases show that dysgeusia may appear earlier than typical MG symptoms, and may also occur at the early stage of a myasthenic crisis in the course of MG. Its appearance is related to AChR-Ab titer. Therefore, awareness and alertness to this symptom may help in the early detection and diagnosis of MG, and may even predict relapse or signal the occurrence of myasthenia crisis.

## Supplementary Explanation

1. Taste strips method: it is based on filter paper strips with four different concentrations of different tastes (including sweet, sour, savory, and bitter). Strips were placed on the outer one-third of the tongue, to either the left or the right. Multiple sites were tested on both sides. A total of 32 tests were performed. Patients were required to determine the taste from a four-descriptor list, and a score was given according to the number of tastes that could be correctly identified by the patient.
2. All AChR-Ab titers in this study were tested by ELISA; the reference value was <0.45 nmol/L.
3. Abbreviations
  SFEMG: single-fiber electromyography
  RNS: repetitive nerve stimulation
  q.d.: quaque die
  t.i.d.: three times a day.

## Ethics Statement

Patient consent: the principle author, FH, has been the primary doctor for these three patients for several years and obtained full consent from these patients to publish the case. Author statement: this study was carried out by the above-mentioned authors. The patients provided their written informed consents.

## Author Contributions

KZ, JC, and HF contributed to the conception and design of the study. All authors have made substantial contributions to the conception, acquisition, analysis, and interpretation of the manuscript and critically revised the manuscript for intellectual content and have given their approval for the final version to be published.

## Conflict of Interest Statement

The authors declare that the research was conducted in the absence of any commercial or financial relationships that could be construed as a potential conflict of interest.
